# The American Dental Association

**Published:** 1863-06

**Authors:** John F. Johnston


### Correspondence.

### THE AMERICAN DENTAL ASSOCIATION.

The regular annual meeting of this Association takes place in the city of Philadelphia upon the last Tuesday of July next, at ten o'clock A.M. All journals favorable to the advancement of dental science will confer a favor upon the Association, by publishing this notice.

JOHN F. JOHNSTON,
*Cor. Secretary.*

*A few words to the Profession Generally:*

The Corresponding Secretary entertaining the opinion that all members of the American Dental Association as well as delegates elect, and all others of the profession, who would have, or be likely to take, any interest in it, will see the above notice in at least some one of our dental periodicals, will, in the absence of definite instructions, omit sending circulars as has been done heretofore. Being also fully convinced that this will be one of the most useful and important meetings the Association will have held, he will, at the risk of being thought tedious and dictatorial, venture to remind the profession generally and delegates of the various Dental Societies *particularly* that it is their plain duty, and will be decidedly to their advantage, to be present. He is assured that they will be received by their prefessional brethren in Philadelphia with an unaffected cordial greeting; indeed, he knows this from experience, having been the happy recipient of their kindness during a very brief visit last summer. But aside from their known hospitality, the many places of interest in the beautiful city—Dr. S. S. White's dental depot, for instance where those who live at remote points from dental furnishing establishments will find themselves repaid for the journey in the facilities offered in obtaining dental goods; aside from these, and many other outside advantages, and above them all, this Association ought to be encouraged by full attendance of its members, and all others interested in substantial scientific advancement, which this Association from the nature of its organization and working rules is perhaps better able to accomplish than any other if properly sustained. Although society is much disturbed by the terrible troubles of the nation, yet money is abundant and the business in the aggregate has suffered but little. But even if it does require unusual effort, it is yet earnestly hoped that the meeting in the beautiful city of "steady habits and brotherly love" will be well attended and productive of much good.

JOHN F. JOHNSTON,
*Cor. Sec'y, Am. Dental Association.*

